# Novel Ribonuclease Activity Differs between Fibrillarins from *Arabidopsis thaliana*

**DOI:** 10.3389/fpls.2017.01878

**Published:** 2017-10-31

**Authors:** Ulises Rodriguez-Corona, Alejandro Pereira-Santana, Margarita Sobol, Luis C. Rodriguez-Zapata, Pavel Hozak, Enrique Castano

**Affiliations:** ^1^Unidad de Bioquímica y Biología Molecular de Plantas, Centro de Investigación Científica de Yucatán, Mérida, Mexico; ^2^Biosystematics Group, Department of Plant Sciences, Wageningen University and Research, Wageningen, Netherlands; ^3^Department of Biology of the Cell Nucleus, Institute of Molecular Genetics of the Academy of Sciences of the Czech Republic, Prague, Czechia; ^4^Unidad de Biotecnología, Centro de Investigación Científica de Yucatán, Mérida, Mexico

**Keywords:** nucleoli, fibrillarin, ribonuclease, phosphoinositides, phosphatidic acid, glycine-arginine rich domain

## Abstract

Fibrillarin is one of the most important nucleolar proteins that have been shown as essential for life. Fibrillarin localizes primarily at the periphery between fibrillar center and dense fibrillar component as well as in Cajal bodies. In most plants there are at least two different genes for fibrillarin. In *Arabidopsis thaliana* both genes show high level of expression in transcriptionally active cells. Here, we focus on two important differences between *A. thaliana* fibrillarins. First and most relevant is the enzymatic activity by AtFib2. The AtFib2 shows a novel ribonuclease activity that is not seen with AtFib1. Second is a difference in the ability to interact with phosphoinositides and phosphatidic acid between both proteins. We also show that the novel ribonuclease activity as well as the phospholipid binding region of fibrillarin is confine to the GAR domain. The ribonuclease activity of fibrillarin reveals in this study represents a new role for this protein in rRNA processing.

## Introduction

The nuclear architecture and gene regulation are some of the most relevant subjects in science today. During the last few decades, the study of the molecules involved in gene regulation has revealed several proteins, DNA and RNA as the main players. Recently, other smaller molecules like phospholipids also play a crucial process in the dynamic architecture and function of the nucleus (Sobol et al., 2013; Yildirim et al., 2013). Here we focus on the nucleoli as one of the most studied nuclear structures in eukaryotic cells. Besides ribosomal RNA (rRNA) production and ribosome pre-assembly the nucleolus is also involved in many relevant aspects of the cell life including biogenesis of small nuclear and nucleolar RNA (snRNA and snoRNA, respectively), sensing cellular stress, nucleolar dysfunctions as cancer, genetic silencing, cell cycle, and viral infection progression, senescence among others (Jacobson and Pederson, 1998; Cockell and Gasser, 1999; Garcia and Pillus, 1999; Hernandez-Verdun et al., 2010; Olson and Dundr, 2015). In plants, the nucleolus consists of four components: FC, DFC, GC, and NV. Fibrillarin was first identified in fibrillar and granular regions of the nucleolus with autoimmune sera from a patient with scleroderma(Ochs et al., 1985). Also fibrillarin in plants was detected for first time in onion cells in the transition zone between the FC and the DFC (Cerdido and Medina, 1995). Ultrathin sections of rat neurons have shown fibrillarin localization at the periphery of FC and in the DFC (Desterro et al., 2003). Fibrillarin is a conserved *S*-adenosyl-L-methionine-dependent methyltransferase which is found in all eukaryotic cells and a shorter version exists in the Archaea kingdom as well (Rodriguez-Corona et al., 2015; Shubina et al., 2016). Therefore the only activity assigned to fibrillarin has been methylation of rRNA and histone H2A (Tollervey et al., 1993; Tessarz et al., 2014; Loza-Muller et al., 2015). However, this activity is not essential for life, while fibrillarin is an essential protein in eukaryotic organism so its precise role may still need to be defined. Reduced levels of fibrillarin in *Drosophila melanogaster* exposed to mTOR resulted in lifespan prolongation and a decrease of the nucleolar size in the fat body and intestine cells (Tiku et al., 2016). Since mTOR also regulates p53 and higher levels of p53 directly reduce the amount of fibrillarin. It correlates well with several types of cancers that show the reduction of p53 and therefore an increase of fibrillarin and higher level of methylation in ribosomes causing errors during translation (Marcel et al., 2013). Human fibrillarin also forms a sub-complex with splicing factor 2-associated p32 with unknown function but independent from ribosomal processing (Yanagida et al., 2004). Furthermore, it was surprising that silencing of fibrillarin in human cells shows nuclear structure alterations in a cell cycle dependent manner before the cells death (Amin et al., 2007).

Fibrillarin in plants has been found in pulldowns of the RNA pol II transcription mediator complex as subunit 36a. *Arabidopsis thaliana* has three different genes of fibrillarin (Barneche et al., 2000). It is also involved in the viral progression and long distance trafficking of viruses in plants like the Bamboo mosaic potexvirus satRNA forms a ribonucleoprotein complex with fibrillarin and this complex allows the virus phloem based movement and infection in other tissues (Chang et al., 2016). Due to the several unknowns of this protein, we therefore decided to study both fibrillarin proteins: fibrillarin 1 (AtFib1) and fibrillarin 2 (*FLP* fibrillarin-like protein; AtFib2) from *A. thaliana* as a model plant. In most eukaryotic cells, fibrillarin localizes primarily in the FC and DFC regions of the nucleolus, where active ribosomal DNA (rDNA) transcription and rRNA processing takes place. Both proteins contain three domains; glycine-arginine rich domain (GAR domain), methyltransferase domain and alpha region. The domains are very well conserved with the exception of the GAR domain that does not exist in the Archaea. The GAR domain has been shown to be required for nucleolar localization of fibrillarin (Snaar et al., 2000), but no further studies have been carried out on the function of this domain. In human cells, recent work demonstrated how two nucleolar proteins, fibrillarin and nucleophosmin, can phase-separate into droplets similar to the subnucleolar compartments *in vitro* and *in vivo* (Feric et al., 2016). This is attributed to the physical properties of the GAR domain resulting in a disordered structure in fibrillarin. However, the combination sequence of GAR domain and at least one RRM of fibrillarin is required for proper subnucleolar compartment formation and maintenance (Feric et al., 2016).

In the last few years, questions as to the endonuclease activity required for the proper processing for rRNA has shown to involve a complex were several proteins are, including fibrillarin (Henras et al., 2015). In yeast depleted U3 snoRNA causes affect knob formation on nascent pre-rRNA and alter as seen on the promoters by electron microscopy (Dragon et al., 2002). During our studies throughout purifications we discover that fibrillarin has a ribonuclease activity, here we show a distinction on this activity between the two fibrillarins of *A. thaliana.* Furthermore, in this study we show the interaction of both fibrillarins with phosphoinositides, which is involved in several nuclear functions (Sobol et al., 2013; Yildirim et al., 2013), and therefore may provide clues for uncovering the fibrillarin nuclear dynamics.

## Materials and Methods

### Bioinformatic Analysis

**FIGURE 1:**
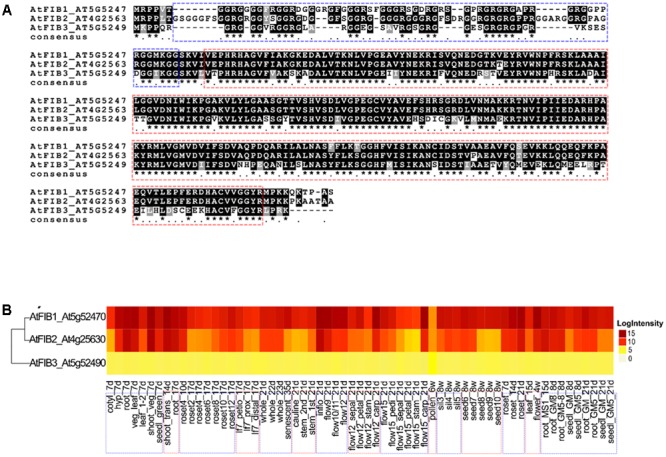
Alignment and ribonuclease activity of *A. thaliana* fibrillarins. **(A)** Sequence comparison of the three AtFib proteins by aligning in MAFFT program. **(B)** Heat-map of the transcriptional expression patterns of AtFib1-3 genes in different tissues and different developmental stages in wild-type *Arabidopsis* Col-0. Data were taken from (Schmid et al., 2005).

Amino acid alignment in **Figure 1A** was visualized by BOXSHADE v3.3.1C^1^. Gene expression data for AtFib proteins in **Figure 1B** was taken from Schmid et al. (2005). Treatment descriptions and gene expression information can be inspected in TA°IR (accession: ExpressionSet: 1006710873), and also can be inspected in http://www.PLEXdb.org (accession: AT40). The heatmap in **Figure 1B** was generated by using the ComplexHeatmap package (Gu et al., 2016) from Bioconductor project (Huber et al., 2015). Structural studies of *Arabidopsis* fibrillarins were modeled on the free software 3d-jigsaw^2^ and edited with PyMOL v1.8.4.0.

### Cloning

*Arabidopsis thaliana* plants were cultivated on soil in a controlled environment and photoperiod of 10–13 h light at 23°C and 11–14 h dark ^∗∗∗^at 20°C (Yoo et al., 2007). RNA extraction was made with RNeasy^®^ Plant Mini Kit (QIAGEN Sciences, Germantown, MD, United States). Sequences of AtFib1 and AtFib2 were obtained with SuperScript^TM^ III One-Step RT-PCR System with Platinum^TM^ Taq DNA Polymerase (Thermo Fischer Scientific). The specific primers to amplify AtFib1 were: forward 5′ – 3′ CATATGATGAGACCCCCAGTTACAGGA and reverse 5′ – 3′ GGATCCCTATGA GGCTGGGGTCTTTTG. To amplify Atfib2 the specific primers were: forward 5′ – 3′ CATATGATGAGACCTCCTCTAACTGGAAG and reverse 5′ – 3′ GGATCCTCTAAG CAGCAGTAGCAGCCTTTG. Forward primers have NdeI restriction enzyme sequence, reverse primers have BamHI restriction enzyme sequence for pET15b expression plasmid cloning. Same strategy was used for GAR (AtGAR2) and alpha helix (Atα2) domain cloning of AtFib2. AtGAR2 primers: forward: 5′ – 3′ CATATGATGAGACCTCCTCTA ACTGGAAG, reverse 5′ – 3′: GGATCCCACAATCACTTTGCTTCCTCC. Atα2 primers: forward 5′ – 3′: CATATGCTTGTAGGCATGGTTGATGT, reverse 5′ – 3′: GGATCC CAAAGGCTGCTACTGCTGCTTAG.

### Protein Expression and Purification

*Arabidopsis thaliana* fibrillarins were expressed in *E. coli* Artic competent cells induced with 1 mM isopropyl-D-1-thiogalactopyranoside at 11°C for 24 h. Harvested cells were suspended in protein extraction buffer (500 mM NaCl, 25 mM tris pH 8, 10% glycerol, 20 mM imidazole, 0.1% triton X-100, 0.1 mM AEBSF and 0.1 mM DTT) and sonicated. After clarification by centrifugation (17400 × *g* × 15 min), the supernatant was subjected to further purification steps. The clarified supernatant was loaded onto a Ni-NTA agarose column (Thermo Fisher Scientific) and washed three times with the extraction buffer. Fibrillarins were eluted (200 mM NaCl, 25 mM tris pH 8, 20% glycerol, 0.1 mM AEBSF and 0.1 DTT) in a linear gradient from 20 to 200 mM of imidazole. Fibrillarins containing fractions were further purified by Q sepharose chromatography leading to single band detection of fibrillarins. Same strategy was used for AtGAR2 and Atα2 domains.

### *In gel* RNase Activity

Proteins were separated in 15% SDS-PAGE gel. Prior to polymerization, running gel was supplied with 5 mg/mL of total RNA extracted from *A. thaliana*. After electrophoresis, gel was washed for 10 min with buffer I (10 mM Tris-HCl, 20% isopropanol, pH 7.5) and consequent incubation for 30 min in buffer II (10 mM Tris-HCl, pH 7.5) and buffer III (100 mM Tris-HCl, pH 7.5). Gel was stained with 0.2% of toluidine blue and washed with water (Dudkina et al., 2016).

### *In Vitro* Transcription

*Arabidopsis thaliana* snoRNA U3 sequence was amplified and cloned into pGEM-T^®^ Easy Vector (PROMEGA). Once cloned, vector was linearized with NdeI enzyme for 1h at 37°C. Transcription was made with T7 RNA polymerase (New England Biolabs Inc.) for 2 h at 37°C. Specific primers for AtsnoU3 used are: forward 5′–3′ ACGACCTTACTTGAACAGGA, reverse 5′–3′ CCTGTCAGACCGCCGTGC GAC.

### Ribonuclease Assay

Total RNA extracted from *A. thaliana* was mixed with each fibrillarin on BC200 buffer (20 mM Tris-HCl buffer, pH 8, 200 mM KCl, 0.2 mM EDTA, 10% glycerol), incubated for 1 h at 37°C and then loaded in a 1% agarose gel.

### Fat Blot Assay

PIP strip with spotted phosphoinositides (Echelon Biosciences, P-6001) was probed with anti-Fib antibody. For this, the membrane was blocked with 3% BSA in PBS for 1 h followed by 3 h at room temperature of 1% BSA in PBS and 0.4 μg of each protein. After that, PIP strip was washed three times, 10-min each, with PBS-T and incubated with primary antibody for 1 h. Again washed with PBS-T and incubated with the appropriate IRDye secondary antibody for 1 h. The immunoblotting signals were analyzed by Odyssey Infrared Imager 9120 (LI-COR Biosciences, Lincoln, NE, United States).

### Western Blot Assay

Fifty nanograms of each fibrillarin was loaded in a 12% acrylamide gel to perform a SDS-PAGE. Subsequently we transfer the protein to a nitrocellulose membrane and blocked with 3% of BSA in PBS at room temperature. Later was made incubation with anti-Fib rabbit antibody (1/5000) for 2 h at room temperature and a third with secondary antibody (1/4000) 1 h at room temperature, with three washes between incubations and revealed. The immunoblotting signals were analyzed by Odyssey Infrared Imager 9120 (LI-COR Biosciences, Lincoln, NE, United States).

### Immunofluorescence

*Arabidopsis thaliana* callus were made according to Sugimoto and Meyerowitz (2013). Sample preparations for microscopy analysis was made as previously publish by our group (Loza-Muller et al., 2015) with callus from *A. thaliana* instead of leaves. Images were acquired in confocal microscope (Leica TCS SP5 AOBS TANDEM) and a laser-scanning microscope FV100 Olympus with 60X (NA 1.4) oil immersion objective lens.

## Results

**FIGURE 2:**
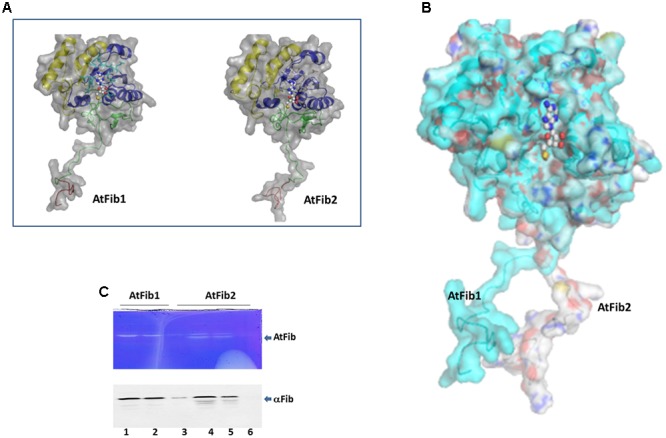
Structural difference between fibrillarins from *A. thaliana.* **(A)** The domains are shown as follows. In Red the GAR domain, Green: BCO space region, Yellow: RNA binding domain, Blue: Alpha helix domain. SAM is showed as spheres. The molecular surface of the proteins is showed in gray color. **(B)** Structural alignment of AtFib1 and AtFib2. The alignment shows that GAR domain and BCO space region are oriented in opposite directions in these two proteins. **(C)** *In gel* ribonuclease activity assay. The white bands correspond to the spaces in the gel, in which RNA was degraded by fibrillarin, confirmed by Western blot. 1, 2, and 3 are three different elutions from the purification process of AtFib1. 4, 5, and 6 are three different elutions from the purification process of AtFib2.

The comparison between the three fibrillarin genes in *A. thaliana* shows the greater amino acid difference in the GAR domain represented by a dotted blue contour (**Figure 1A**). Considering that this part of the protein resides the main difference we check if their expression would be tissue or developmental stage specific. Transcriptional patterns of AtFib genes (**Figure 1B**) demonstrate high expression levels in the different tissues and on different developmental stages in wild-type *Arabidopsis* Col-0 (data can be inspected in http://www.PLEXdb.org, accession: AT40). Sequence differences between AtFib proteins (GAR domain + fibrillarin domain) could have major implications on protein activity. At transcriptional level, we found differences between AtFib genes. AtFib1 shows the highest expression levels but also little variations between treatments, while AtFib2 shows the most variation between treatments and seems to be more affected by development stages but remains expressed in all stages. AtFib3 shows the lowest level of expression values and almost no variation between tissues and development stages. We focused on AtFib1 and AtFib2 as they are expressed in almost all conditions. The *in silico* structure prediction between them (**Figure 2A**) shows that the main structural difference is due to an angle changed for the exposure of the GAR domain as can be seen in the overlay of the structures in **Figure 2B**. As in other crystal structure of fibrillarin (Rodriguez-Corona et al., 2015), the regions of methyl transferase to alpha region maintain a similar structure.

Our initial studies where to test gel mobility alterations by fibrillarin with RNA resulted in degradation of the RNA when a short incubation was carried out at room temperature. We therefore tested the purified fibrillarin with an *in gel* ribonuclease activity assay to make sure that no other protein was responsible for this activity. The *in gel* toluidine blue staining of RNA show a white band from the lack of RNA due to its degradation at the correct molecular weight for the purified fibrillarin (**Figure 2C**). Different eluates were loaded in the ribonuclease activity gel assay and show that both fibrillarins (AtFib1 and AtFib2) have ribonuclease activity. Western blot of the bands confirmed their correspondence to fibrillarin (**Figure 2C**). AtFib2 is more susceptible to degradation as showed by Western blot and as *in gel* activity assay.

**FIGURE 3:**
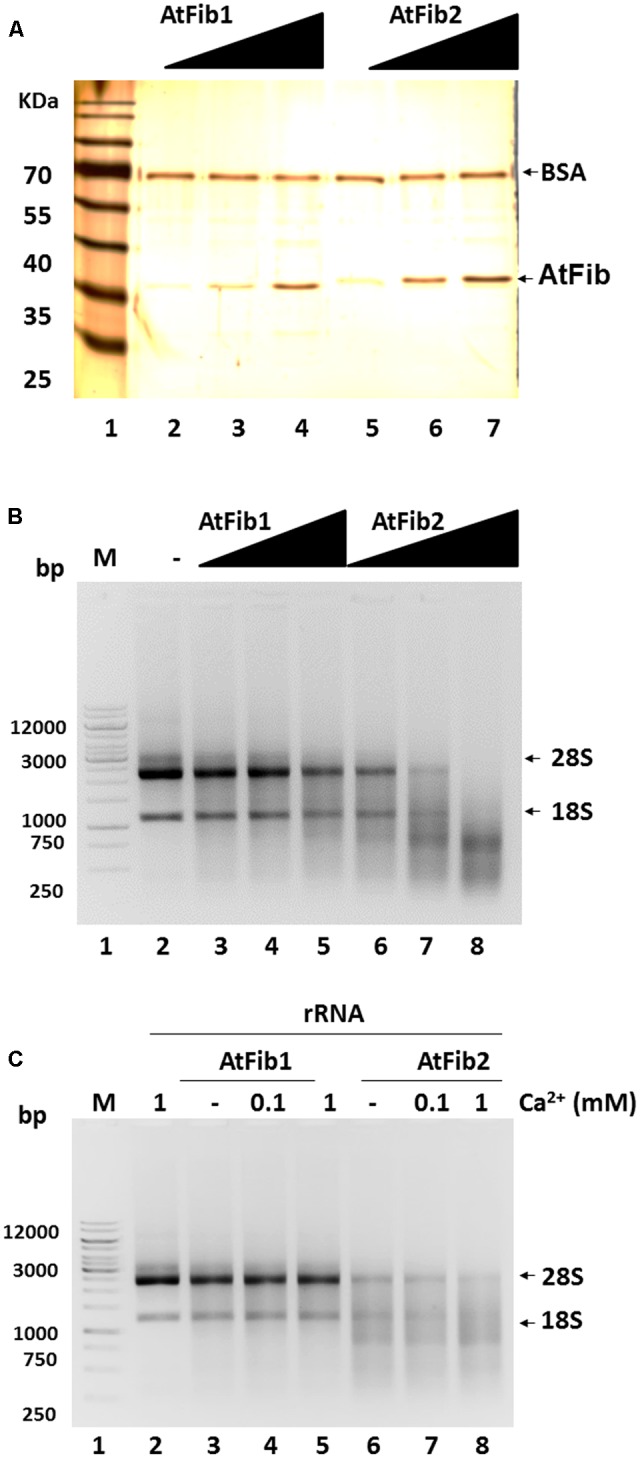
Ribonuclease activity of *A. thaliana* fibrillarins in calcium presence. **(A)** Concentration from 2 to 8 ng of both *A. thaliana* fibrillarins. BSA was added to normalize. **(B)** Fibrillarin ribonuclease activity. Increased amounts of fibrillarin were added to a constant concentration of rRNA. The assay clearly shows that AtFib1 is less active as compared to AtFib2 at the same concentration. **(C)** Using the concentration of fibrillarins as shown in lane 3 and 5 of **Figure 3A**, we tested further the activation of ribonuclease activity by calcium for AtFib1 and AtFib2 (lane 4 – 5 and 7 – 8, respectively). Calcium was added at the concentrations of 0.1–1 mM.

We decided to characterize this novel ribonuclease activity and purified both proteins to homogeneity (**Figure 3A**) in the exact same procedure and tested their activity under native conditions. Both fibrillarins were incubated with rRNA to test their ability to cleave rRNA. The reactions were carried out using the same amounts of fibrillarins as what is shown in the silver stained gel (**Figure 3A**). The results shown in **Figure 3B** demonstrate that AtFib2 has a potent ribonuclease activity in a dose dependent manner while AtFib1 can only show activity under the greatest amount. This correlates well with the *in gel* activity assay which shows both proteins to have activity but AtFib2 show significant rRNA cleavage. We tested if *A. thaliana* fibrillarins are activated by calcium, as other ribonucleases (Schwarz and Blower, 2014), we found that AtFib1 is not activated by calcium, while AtFib2 shows minor activation (**Figure 3C**). Interestingly, the activation of the ribonuclease activity by calcium shown for AtFib differs from that of human fibrillarin that we tested (data not shown).

**FIGURE 4:**
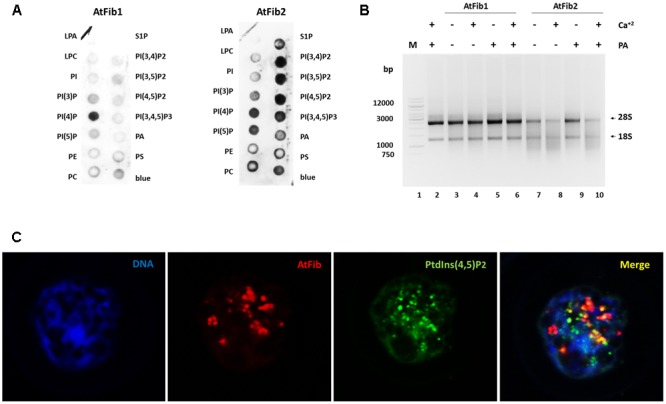
*Arabidopsis thaliana* fibrillarins and phosphoinositides. **(A)** Fat blot assay for AtFib1 and AtFib2. AtFib1 interacts mainly with the monophosphate phosphoinositides, in contrast AtFib2 interacts with all phosphoinositides and phosphatidic acid. **(B)** Ribonuclease activity in phosphatidic acid presence. With the same concentration for both fibrillarins as **Figure 3A**, lanes 3 and 5, its clear how in phosphatidic acid presence (30 ng) the ribonuclease activity of AtFib2 is inhibit (lane 9). **(C)** Colocalization between AtFib’s and PtdIns(4,5)P_2_ in *A. thaliana* callus.

Our previous studies with human fibrillarin had shown its interaction with phosphoinositides (Yildirim et al., 2013). In **Figure 4A**, AtFib1 primarily interacts with PtdIns(4)P, while AtFib2 interacts with all phosphoinositides, as well as with phosphatidic acid (PA). This is similar to what we have detected with the unique human fibrillarin (Yildirim et al., 2013 and data not shown). PA is implicated in many stress events in plants and it is also involved in phosphoinositides metabolism. Here, we detected a decrease of the ribonuclease activity by the addition of PA as seen in **Figure 4B**, lane 9. PA inactivation is reversed by the addition of calcium (**Figure 4B**, lane 10).

Nuclear phosphoinositides have been extensively studied in plant membranes but studies are lacking on the nuclear forms. To provide more information on nuclear phosphoinositides, we took advantage of the PtdIns(4,5)P_2_ antibody. We carried out confocal microscopy of *Arabidopsis* callus which had membrane bound PtdIns(4,5)P_2_ removed by Triton X-100 as it was done in other publications (Laboure et al., 1999) (**Figure 4C**). We show that nuclear PtdIns(4,5)P_2_ has a partial colocalization with fibrillarin. Since the antibodies against fibrillarin detect both forms of fibrillarin it is impossible to discern between the two forms at this stage. We have unsuccessfully tried to raise antibodies, which would distinguish between these two fibrillarins that may lead to a better colocalization of one of them with phosphoinositides. The PtdIns(4,5)P_2_ exhibits a dotted pattern in nucleoli regions and a diffuse pattern in other nuclear regions. Fibrillarin colocalizes with PtdIns(4,5)P_2_ in the nucleolus but not in other regions like Cajal bodies.

**FIGURE 5:**
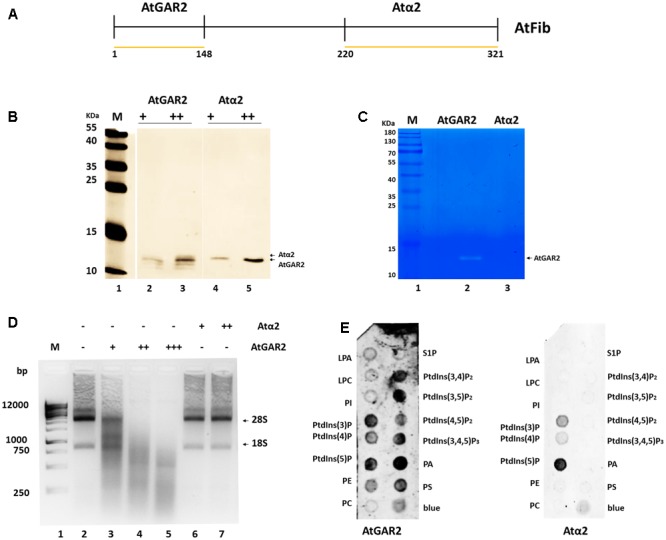
Ribonuclease activity of AtFib2 domains. **(A)** Schematic representation of AtFib. Yellow lines represent the expressed domains [AtGAR2 (1–48 amino acids) and Atα2 (220–321 amino acids)]. **(B)** Western blot for AtFib2 domains. Two different concentrations of AtGAR2 and Atα2 were recognized with anti-HIS primary antibody. **(C)** *In gel* ribonuclease activity assay. The white bands correspond to the spaces in the gel in which RNA was degraded by AtGAR2. **(D)** Ribonuclease activity of AtGAR2 and Atα2 domains. Degradation of rRNA was directly related to the amount of AtGAR2 domain added. **(E)** Fat blot assay for AtGAR2 and Atα2. AtGAR2 domain interacts with all phosphoinositides in the same way as the whole AtFib2 protein. By the other hand, Atα2 interacts mainly with PtdIns(5)P.

**FIGURE 6:**
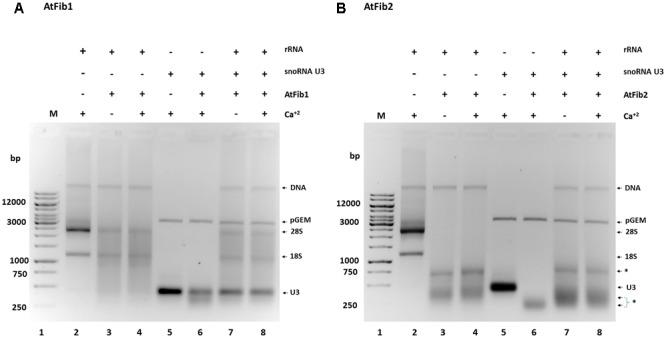
Ribonuclease activity against rRNA and snoRNA U3. **(A)** AtFib1 ribonuclease activity against rRNA and snoRNA U3. As expected, compared to AtFib2, AtFib1 has significantly lower ribonuclease activity either to rRNA (lanes 3 and 4) or to snoRNA U3 (lane 6–8). **(B)** AtFib2 ribonuclease activity against rRNA and snoRNA U3. As expected, compared to AtFib1, AtFib2 has ribonuclease activity against rRNA (lanes 3 and 4) and snoRNA U3 (lane 6–8). The asterisks show ^∗^ the positions of specific RNA bands originated from RNAs cleaved by AtFib2.

In order to define the domain that has ribonuclease activity, we overexpressed two domains of the protein, which were shown to have an enzymatic activity assigned (**Figure 5A**). The N terminus contains the GAR domain and the C terminus the α domain. We purified both domains (**Figure 5B**) and tested them for activity. Only AtGAR2 domain showed high ribonuclease activity both in an *in gel* activity assay with RNA as substrate, as well as under native conditions (**Figures 5C,D**). The ribonuclease activity of AtGAR2 domain is less selective than the full fibrillarin protein as it degrades both 28S and 18S simultaneously and gives a less selective pattern of bands as well; **Figure 5D**, lanes 4 and 5. The AtGAR2 domain is also the interacting domain for phospholipid binding, including all phosphoinositides species as well as PA and phosphatidylserine (PS) and resemble the full protein binding, while the alpha region of AtFib2 had no ribonuclease activity and only binds to PtdIns(5)P (**Figure 5E**). Finally, we compared AtFib1 and AtFib2 for their ribonuclease activity on U3 guide RNA and overall rRNA. We found that AtFib2 was able to cut RNA as compared between **Figures 6A,B**. AtFib1 showed only a minor reduction in the amount of rRNA but maintained the exact same pattern, while AtFib2 showed a different pattern of rRNA and U3 after interacting with this fibrillarin as seen in **Figure 6B**, lane 6 compared to lanes 7 and 8.

## Discussion

The plants genomes are a mix of duplicated and triplicated regions, which results from the series of whole-genome duplication events (WGD) known as paleopolyploidy, events that occurred throughout plant evolution. These events have played a major role in Brassicaceae evolution. *A. thaliana* has undergone three paleopolyploidy events (At-α, At-β, and At-γ; Bowers et al., 2003; Schranz et al., 2012). These variations in gene copy number, retention of duplicated copies, and posterior sub- or neo-functionalization, increase the genetic variation (van den Bergh et al., 2016) which play an essential role in the environment adaptation (Dassanayake et al., 2011). The major transcriptional differences between AtFib genes indicate the great importance of the functional fate of duplicated copies, which could have implications on protein activity. AtFib1 and AtFib2 are expressed in large amounts and in all tissues as seen in **Figure 1B**. Therefore, changes in the known functions can be expected for these proteins as they acquire different mutations. However, the differences are localizing to the GAR domain. Fibrillarin is also well known to be involved in pre-rRNA processing in nucleolus in several organisms. However, the mechanism of its action is still largely unknown and a variation of function may occur during gene duplication and subsequent differential mutagenesis. Since the early experiments of Tollervey et al. (1993) with temperature sensitive mutants of Nop1 (yeast fibrillarin), the main attributed activity of fibrillarin was a methyltransferase for rRNA and more recent for histone H2A (Tessarz et al., 2014; Loza-Muller et al., 2015). However, even during the early experiments with mutant Nop1, the yeasts showed different phenotypes before dying at the non-permissive temperature, in particular, the nop1.2 and nop1.5 alleles showed a reduced level of synthesis for both 18S and 25S rRNA, moreover the production of all pre-rRNA species decreased except the main 35S primary transcript (Tollervey et al., 1993). This indicates that some mutants are not able to cut the pre-RNA to produce the mature forms.

One of the main features of fibrillarin is the N-terminal GAR domain. It is the least evolutionary conserved domain of the protein; however, this sequence was added in the transition between Archaea to eukaryotic cells as it is absent in all archaebacteria. This domain is also responsible for the phosphoinositide binding, which well correlates with the lack of it in Archaea kingdom (Amiri, 1994; Hickey et al., 2000; Wang et al., 2000). Furthermore, nucleolar localization requires the GAR domain (Snaar et al., 2000). Fibrillarin forms a complex with Nop56, Nop58, a guide RNA and 15.5k, we postulate that the fibrillarin ribonuclease activity is directed by the complex to selective sites. Currently, we and others have been unsuccessful to form an active eukaryotic ribonucleoprotein complex with fibrillarin (Peng et al., 2014). These complexes have been successfully carried out in Archaea that lack the GAR domain, but not with any of the eukaryotic counterparts (Peng et al., 2014).

One elusive question in regard to ribosomal processing is the nature of the endonuclease activity involved in catalysis of the primary pre-RNA cleavage in eukaryotic cells. Fractions carried out by Saez-Vasquez et al. (2004) showed a highly purified high-molecular-weight complex, which reproduce this cleavage *in vitro*. The authors could not discern which protein had the ribonuclease activity, but they identified nucleolin and fibrillarin as important proteins in this fraction (Saez-Vasquez et al., 2004). Other previous experiments suggested that fibrillarin is the ribonuclease protein involved in the cleavage of rRNA (Kass et al., 1990). They used specific antibodies against human fibrillarin native complex in an *in vitro* ribonuclease assay and showed a decrease in activity when the fibrillarin was blocked (Kass et al., 1990). Surprisingly the authors did not suggest that fibrillarin was involved in the cleavage of rRNA but assumed that it affected the complex. Also fibrillarin was identified in the classical RNA spreads during ribosomal transcription shown as “Christmas trees” as part of the pre-rRNA early processing complexes (Scheer and Benavente, 1990). From our work, we can speculate that AtFib2 ribonuclease activity is involved in the processing of rRNA and that when complex with Nop 56, 58, and 15.5K together with the guide RNA may direct fibrillarin for sequence specific breaks as was shown with the complex by Kass et al. (1990).

Previously we showed that human fibrillarin was able to interact with PtdIns(4,5P)_2_, one of seven phosphoinositides (Yildirim et al., 2013). Amino acids 9–25 of the GAR domain of both Atfib2 and human fibrillarin are absent in Atfib1 and may explain their similarities between both of these proteins. The nuclear phospholipids, in particular phosphoinositides, can be located in nuclear speckles, intra nuclear chromatin domains as well as nucleoli. They interact with a wide range of proteins like: Star-PAP poly(A) polymerase, histone 1, TAF3, UBF, etc. (Osborne et al., 2001; Yildirim et al., 2013; Divecha, 2016). The interaction of phospholipids with such proteins can result in the activation of the protein (like Start-PAP) or affect the stability with other proteins to form particular complexes like TAF3 with H3K4me3 (Stijf-Bultsma et al., 2015). The complex nuclear environment contains large amounts of these phospholipids in a non-membrane fashion for complex formation.

Here, we show a differential binding of phospholipids to *A. thaliana* fibrillarins. Taken into account that phosphoinositides–protein interaction affects the protein ability to form new complexes it is therefore likely that both fibrillarins in *A. thaliana* bind to different partners. This may also explain why confocal microscopy of both fibrillarins does not colocalize 100% with the PtdIns(4,5)P_2_ signal as it does in human cells (Sobol et al., 2013). PA has been shown to inhibit RNase A (Hatton et al., 2015), here we show that it is also able to decrease the ribonuclease activity of fibrillarin.

It has been proposed that GAR domain can destabilize the RNA secondary structure during their interaction (Pih et al., 2000). However, it is unclear which structure can be generated when GAR domain is bound to phospholipids or during its interaction with RNA. The interaction of GAR domain with phospholipids may also explain the fibrillarin phase separation behavior for proper subnucleolar compartment formation and maintenance (Feric et al., 2016). However, the structural phase separation may be more complex involving phospholipids and their metabolism, as well as other ribonucleoproteins and guides RNA. The structure alterations of the nucleoli can be observed with different transcription inhibitors like actinomycin D. Upon transcription inhibition, the separation of nucleolar compartments forms a two phase separated system similar to what is observed whit a mix of hydrophobic molecules in water (Sobol et al., 2013; Feric et al., 2016).

Several questions arise from this work including the role of fibrillarin in Cajal bodies: does it have a role in mRNA processing? Is there a ribonuclease role of fibrillarin as mediator 36a? During cell cycle, does the alteration in nuclear structure in fibrillarin depleted cells is due to degradation of structural RNA? Do viral particles require fibrillarin due to its role in RNA processing? Does GAR domain methylation by any or all of the methyltransferases (PRMT1, PRMT3, PRMT5, etc.) affect ribonuclease activity?

## Author Contributions

UR-C: Experiments design and conception and wrote the article. AP-S: Bioinformatics and wrote the article. MS: Microscopy related experiments. LR-Z: Experiments design and article discussions. PH: Article discussions and review. EC: Experiment design and original set of experiments that led to the research, article writing, and revisions.

## Conflict of Interest Statement

The authors declare that the research was conducted in the absence of any commercial or financial relationships that could be construed as a potential conflict of interest.

## Footnotes

1 http://boxshade.sourceforge.net/

2 https://bmm.crick.ac.uk/3djigsaw/

## Abbreviations

AtFib: *A. thaliana* fibrillarin
DFC: dense fibrillarin component
FAA: formalin-acetic acid-alcohol
FC: fibrillar center
GAR: glycine arginine rich domain
GC: granular component
NV: nucleolar vacuole
PtdIns(4)P: phosphatidylinositol 4-phosphate
PtdIns(4,5)P_2_: phosphatidylinositol 4,5-bisphosphate
RRM: RNA recognition motif

## References

[B1] Amin M. A. Matsunaga S. Ma N. Takata H. Yokoyama M. Uchiyama S. (2007). Fibrillarin, a nucleolar protein, is required for normal nuclear morphology and cellular growth in HeLa cells. *Biochem. Biophys. Res. Commun.* 360 320–326. 10.1016/j.bbrc.2007.06.092 17603021

[B2] Amiri K. A. (1994). Fibrillarin-like proteins occur in the domain Archaea. *J. Bacteriol.* 176 2124–2127. 10.1128/jb.176.7.2124-2127.1994 8144483 PMC205323

[B3] Barneche F. Steinmetz F. Echeverria M. (2000). Fibrillarin genes encode both a conserved nucleolar protein and a novel small nucleolar RNA involved in ribosomal RNA methylation in *Arabidopsis thaliana*. *J. Biol. Chem.* 275 27212–27220. 10.1074/jbc.M002996200 10829025

[B4] Bowers J. E. Chapman B. A. Rong J. Paterson A. H. (2003). Unravelling angiosperm genome evolution by phylogenetic analysis of chromosomal duplication events. *Nature* 422 433–438. 10.1038/nature01521 12660784

[B5] Cerdido A. Medina F. J. (1995). Subnucleolar location of fibrillarin and variation in its levels during the cell cycle and during differentiation of plant cells. *Chromosoma* 103 625–634. 10.1007/BF00357689 7587585

[B6] Chang C. H. Hsu F. C. Lee S. C. Lo Y. S. Wang J. D. Shaw J. (2016). The nucleolar fibrillarin protein is required for helper virus-independent long-distance trafficking of a subviral satellite RNA in plants. *Plant Cell* 28 2586–2602. 10.1105/tpc.16.00071 27702772 PMC5134973

[B7] Cockell M. M. Gasser S. M. (1999). The nucleolus: nucleolar space for RENT. *Curr. Biol.* 9 R575–R576. 10.1016/S0960-9822(99)80359-5 10469557

[B8] Dassanayake M. Oh D. H. Haas J. S. Hernandez A. Hong H. Ali S. (2011). The genome of the extremophile crucifer *Thellungiella parvula*. *Nat. Genet.* 43 913–918. 10.1038/ng.889 21822265 PMC3586812

[B9] Desterro J. M. Keegan L. P. Lafarga M. Berciano M. T. O’connell M. Carmo-Fonseca M. (2003). Dynamic association of RNA-editing enzymes with the nucleolus. *J. Cell Sci.* 116 1805–1818. 10.1242/jcs.00371 12665561

[B10] Divecha N. (2016). Phosphoinositides in the nucleus and myogenic differentiation: how a nuclear turtle with a PHD builds muscle. *Biochem. Soc. Trans.* 44 299–306. 10.1042/BST20150238 26862219

[B11] Dragon F. Gallagher J. E. Compagnone-Post P. A. Mitchell B. M. Porwancher K. A. Wehner K. A. (2002). A large nucleolar U3 ribonucleoprotein required for 18S ribosomal RNA biogenesis. *Nature* 417 967–970. 10.1038/nature00769 12068309 PMC11487672

[B12] Dudkina E. Ulyanova V. Shah Mahmud R. Khodzhaeva V. Dao L. Vershinina V. (2016). Three-step procedure for preparation of pure *Bacillus altitudinis* ribonuclease. *FEBS Open Bio* 6 24–32. 10.1002/2211-5463.12023 27047739 PMC4794795

[B13] Feric M. Vaidya N. Harmon T. S. Mitrea D. M. Zhu L. Richardson T. M. (2016). Coexisting liquid phases underlie nucleolar subcompartments. *Cell* 165 1686–1697. 10.1016/j.cell.2016.04.047 27212236 PMC5127388

[B14] Garcia S. N. Pillus L. (1999). Net results of nucleolar dynamics. *Cell* 97 825–828. 10.1016/S0092-8674(00)80794-1 10399910

[B15] Gu Z. Eils R. Schlesner M. (2016). Complex heatmaps reveal patterns and correlations in multidimensional genomic data. *Bioinformatics* 32 2847–2849. 10.1093/bioinformatics/btw313 27207943

[B16] Hatton N. Lintz E. Mahankali M. Henkels K. M. Gomez-Cambronero J. (2015). Phosphatidic acid increases epidermal growth factor receptor expression by stabilizing mRNA decay and by inhibiting lysosomal and proteasomal degradation of the internalized receptor. *Mol. Cell. Biol.* 35 3131–3144. 10.1128/MCB.00286-15 26124282 PMC4539377

[B17] Henras A. K. Plisson-Chastang C. O’donohue M. F. Chakraborty A. Gleizes P. E. (2015). An overview of pre-ribosomal RNA processing in eukaryotes. *Wiley Interdiscip. Rev. RNA* 6 225–242. 10.1002/wrna.1269 25346433 PMC4361047

[B18] Hernandez-Verdun D. Roussel P. Thiry M. Sirri V. Lafontaine D. L. (2010). The nucleolus: structure/function relationship in RNA metabolism. *Wiley Interdiscip. Rev. RNA* 1 415–431. 10.1002/wrna.39 21956940

[B19] Hickey A. J. Macario A. J. Conway De Macario E. (2000). Identification of genes in the genome of the archaeon *Methanosarcina mazeii* that code for homologs of nuclear eukaryotic molecules involved in RNA processing. *Gene* 253 77–85. 10.1016/S0378-1119(00)00235-3 10925204

[B20] Huber W. Carey V. J. Gentleman R. Anders S. Carlson M. Carvalho B. S. (2015). Orchestrating high-throughput genomic analysis with Bioconductor. *Nat. Methods* 12 115–121. 10.1038/nmeth.3252 25633503 PMC4509590

[B21] Jacobson M. R. Pederson T. (1998). Localization of signal recognition particle RNA in the nucleolus of mammalian cells. *Proc. Natl. Acad. Sci. U.S.A.* 95 7981–7986. 10.1073/pnas.95.14.7981 9653126 PMC20915

[B22] Kass S. Tyc K. Steitz J. A. Sollner-Webb B. (1990). The U3 small nucleolar ribonucleoprotein functions in the first step of preribosomal RNA processing. *Cell* 60 897–908. 10.1016/0092-8674(90)90338-F 2156625

[B23] Laboure A. M. Faik A. Mandaron P. Falconet D. (1999). RGD-dependent growth of maize calluses and immunodetection of an integrin-like protein. *FEBS Lett.* 442 123–128. 10.1016/S0014-5793(98)01634-2 9928986

[B24] Loza-Muller L. Rodriguez-Corona U. Sobol M. Rodriguez-Zapata L. C. Hozak P. Castano E. (2015). Fibrillarin methylates H2A in RNA polymerase I trans-active promoters in *Brassica oleracea*. *Front. Plant Sci.* 6:976. 10.3389/fpls.2015.00976 26594224 PMC4635213

[B25] Marcel V. Ghayad S. E. Belin S. Therizols G. Morel A.-P. Solano-Gonzàlez E. (2013). p53 Acts as a safeguard of translational control by regulating fibrillarin and rRNA methylation in cancer. *Cancer Cell* 24 318–330. 10.1016/j.ccr.2013.08.013 24029231 PMC7106277

[B26] Ochs R. L. Lischwe M. A. Spohn W. H. Busch H. (1985). Fibrillarin: a new protein of the nucleolus identified by autoimmune sera. *Biol. Cell* 54 123–133. 10.1111/j.1768-322X.1985.tb00387.x 2933102

[B27] Olson M. O. Dundr M. (2015). Nucleolus: structure and function. *eLS* 1–9. 10.1002/9780470015902.a0005975.pub3

[B28] Osborne S. L. Thomas C. L. Gschmeissner S. Schiavo G. (2001). Nuclear PtdIns(4,5)P2 assembles in a mitotically regulated particle involved in pre-mRNA splicing. *J. Cell Sci.* 114 2501–2511. 11559758 10.1242/jcs.114.13.2501

[B29] Peng Y. Yu G. Tian S. Li H. (2014). Co-expression and co-purification of archaeal and eukaryal box C/D RNPs. *PLOS ONE* 9:e103096. 10.1371/journal.pone.0103096 25078083 PMC4117494

[B30] Pih K. T. Yi M. J. Liang Y. S. Shin B. J. Cho M. J. Hwang I. (2000). Molecular cloning and targeting of a fibrillarin homolog from Arabidopsis. *Plant Physiol.* 123 51–58. 10.1104/pp.123.1.51 10806224 PMC58981

[B31] Rodriguez-Corona U. Sobol M. Rodriguez-Zapata L. C. Hozak P. Castano E. (2015). Fibrillarin from Archaea to human. *Biol. Cell* 107 159–174. 10.1111/boc.201400077 25772805

[B32] Saez-Vasquez J. Caparros-Ruiz D. Barneche F. Echeverria M. (2004). A plant snoRNP complex containing snoRNAs, fibrillarin, and nucleolin-like proteins is competent for both rRNA gene binding and pre-rRNA processing in vitro. *Mol. Cell. Biol.* 24 7284–7297. 10.1128/MCB.24.16.7284-7297.2004 15282326 PMC479724

[B33] Scheer U. Benavente R. (1990). Functional and dynamic aspects of the mammalian nucleolus. *Bioessays* 12 14–21. 10.1002/bies.950120104 2181998

[B34] Schmid M. Davison T. S. Henz S. R. Pape U. J. Demar M. Vingron M. (2005). A gene expression map of *Arabidopsis thaliana* development. *Nat. Genet.* 37 501–506. 10.1038/ng1543 15806101

[B35] Schranz M. E. Mohammadin S. Edger P. P. (2012). Ancient whole genome duplications, novelty and diversification: the WGD Radiation Lag-Time Model. *Curr. Opin. Plant Biol.* 15 147–153. 10.1016/j.pbi.2012.03.011 22480429

[B36] Schwarz D. S. Blower M. D. (2014). The calcium-dependent ribonuclease XendoU promotes ER network formation through local RNA degradation. *J. Cell Biol.* 207 41–57. 10.1083/jcb.201406037 25287301 PMC4195833

[B37] Shubina M. Y. Musinova Y. R. Sheval E. V. (2016). Nucleolar methyltransferase fibrillarin: evolution of structure and functions. *Biochemistry* 81 941–950. 10.1134/S0006297916090030 27682166

[B38] Snaar S. Wiesmeijer K. Jochemsen A. G. Tanke H. J. Dirks R. W. (2000). Mutational analysis of fibrillarin and its mobility in living human cells. *J. Cell Biol.* 151 653–662. 10.1083/jcb.151.3.653 11062265 PMC2185578

[B39] Sobol M. Yildirim S. Philimonenko V. V. Marasek P. Castano E. Hozak P. (2013). UBF complexes with phosphatidylinositol 4,5-bisphosphate in nucleolar organizer regions regardless of ongoing RNA polymerase I activity. *Nucleus* 4 478–486. 10.4161/nucl.27154 24513678 PMC3925692

[B40] Stijf-Bultsma Y. Sommer L. Tauber M. Baalbaki M. Giardoglou P. Jones D. R. (2015). The basal transcription complex component TAF3 transduces changes in nuclear phosphoinositides into transcriptional output. *Mol. Cell* 58 453–467. 10.1016/j.molcel.2015.03.009 25866244 PMC4429956

[B41] Sugimoto K. Meyerowitz E. M. (2013). Regeneration in *Arabidopsis* tissue culture. *Methods Mol. Biol.* 959 265–275. 10.1007/978-1-62703-221-6_18 23299682

[B42] Tessarz P. Santos-Rosa H. Robson S. C. Sylvestersen K. B. Nelson C. J. Nielsen M. L. (2014). Glutamine methylation in histone H2A is an RNA-polymerase-I-dedicated modification. *Nature* 505 564–568. 10.1038/nature12819 24352239 PMC3901671

[B43] Tiku V. Jain C. Raz Y. Nakamura S. Heestand B. Liu W. (2016). Small nucleoli are a cellular hallmark of longevity. *Nat. Commun.* 8:16083. 10.1038/ncomms16083 28853436 PMC5582349

[B44] Tollervey D. Lehtonen H. Jansen R. Kern H. Hurt E. C. (1993). Temperature-sensitive mutations demonstrate roles for yeast fibrillarin in pre-rRNA processing, pre-rRNA methylation, and ribosome assembly. *Cell* 72 443–457. 10.1016/0092-8674(93)90120-F 8431947

[B45] van den Bergh E. Hofberger J. A. Schranz M. E. (2016). Flower power and the mustard bomb: comparative analysis of gene and genome duplications in glucosinolate biosynthetic pathway evolution in Cleomaceae and Brassicaceae. *Am. J. Bot.* 103 1212–1222. 10.3732/ajb.1500445 27313198

[B46] Wang H. Boisvert D. Kim K. K. Kim R. Kim S. H. (2000). Crystal structure of a fibrillarin homologue from *Methanococcus jannaschii*, a hyperthermophile, at 1.6 A resolution. *EMBO J.* 19 317–323. 10.1093/emboj/19.3.317 10654930 PMC305568

[B47] Yanagida M. Hayano T. Yamauchi Y. Shinkawa T. Natsume T. Isobe T. (2004). Human fibrillarin forms a sub-complex with splicing factor 2-associated p32, protein arginine methyltransferases, and tubulins alpha 3 and beta 1 that is independent of its association with preribosomal ribonucleoprotein complexes. *J. Biol. Chem.* 279 1607–1614. 10.1074/jbc.M305604200 14583623

[B48] Yildirim S. Castano E. Sobol M. Philimonenko V. V. Dzijak R. Venit T. (2013). Involvement of phosphatidylinositol 4,5-bisphosphate in RNA polymerase I transcription. *J. Cell Sci.* 126 2730–2739. 10.1242/jcs.123661 23591814

[B49] Yoo S. D. Cho Y. H. Sheen J. (2007). *Arabidopsis* mesophyll protoplasts: a versatile cell system for transient gene expression analysis. *Nat. Protoc.* 2 1565–1572. 10.1038/nprot.2007.199 17585298

